# PPM1A Controls Diabetic Gene Programming through Directly Dephosphorylating PPARγ at Ser273

**DOI:** 10.3390/cells9020343

**Published:** 2020-02-02

**Authors:** Keon Woo Khim, Sun Sil Choi, Hyun-Jun Jang, Yo Han Lee, Eujin Lee, Ji-Min Hyun, Hye-Jin Eom, Sora Yoon, Jeong-Won Choi, Tae-Eun Park, Dougu Nam, Jang Hyun Choi

**Affiliations:** Department of Biological Sciences, Ulsan National Institute of Science and Technology (UNIST), Ulsan 44919, Korea; kkwzang@unist.ac.kr (K.W.K.); ssili77@unist.ac.kr (S.S.C.); hyunjun86@unist.ac.kr (H.-J.J.); yohan010@unist.ac.kr (Y.H.L.); albina@unist.ac.kr (E.L.); localman@unist.ac.kr (J.-M.H.); eomazing@unist.ac.kr (H.-J.E.); yoonsora1@unist.ac.kr (S.Y.); shappys95@unist.ac.kr (J.-W.C.); tepark@unist.ac.kr (T.-E.P.); dougnam@unist.ac.kr (D.N.)

**Keywords:** obesity, insulin sensitivity, PPM1A, PPARγ S273 phosphorylation, diabetic gene reprogramming

## Abstract

Peroxisome proliferator-activated receptor γ (PPARγ) is a master regulator of adipose tissue biology. In obesity, phosphorylation of PPARγ at Ser273 (pSer273) by cyclin-dependent kinase 5 (CDK5)/extracellular signal-regulated kinase (ERK) orchestrates diabetic gene reprogramming via dysregulation of specific gene expression. Although many recent studies have focused on the development of non-classical agonist drugs that inhibit the phosphorylation of PPARγ at Ser273, the molecular mechanism of PPARγ dephosphorylation at Ser273 is not well characterized. Here, we report that protein phosphatase Mg^2+^/Mn^2+^-dependent 1A (PPM1A) is a novel PPARγ phosphatase that directly dephosphorylates Ser273 and restores diabetic gene expression which is dysregulated by pSer273. The expression of PPM1A significantly decreases in two models of insulin resistance: diet-induced obese (DIO) mice and *db/db* mice, in which it negatively correlates with pSer273. Transcriptomic analysis using microarray and genotype-tissue expression (GTEx) data in humans shows positive correlations between PPM1A and most of the genes that are dysregulated by pSer273. These findings suggest that PPM1A dephosphorylates PPARγ at Ser273 and represents a potential target for the treatment of obesity-linked metabolic disorders.

## 1. Introduction

Obesity, defined as an accumulation of excess body fat, is closely associated with metabolic diseases, such as dyslipidemia, type 2 diabetes, cardiovascular diseases, and certain types of cancer [1,2,3,4]. Adipose tissue plays a pivotal role in excess energy storage and is considered an important regulator of glucose and lipid metabolism, mediated through the secretion of adipokines [5,6,7]. Obesity is characterized by abnormal adipose tissue and the dysregulation of adipokine secretion [8,9,10]. For example, the expression of pro-inflammatory cytokines, such as tumor necrosis factor-α (TNF-α) and interleukin-1β (IL-1β), and chemokines, such as chemokine ligand 2 (CCL2), are higher in obese adipose tissue, whereas the secretion of the insulin-sensitizing adipokines, adiponectin and adipsin, are lower in those tissues [11,12,13,14,15,16,17]. These circulating adipokines specifically orchestrate systemic metabolism by regulating metabolism in peripheral tissues, such as liver, muscle, and macrophages. Thus, an understanding of adipose tissue biology is critical for the most effective treatment of obesity and metabolic diseases.

Peroxisome proliferator-activated receptor gamma (PPARγ) is a nuclear receptor that is a master regulator of adipose tissue [18,19,20]. Indeed, numerous studies show that PPARγ is both necessary and sufficient for fat cell differentiation [21,22,23]. The transcriptional activity of PPARγ is activated by its ligands, which include thiazolidinediones (TZDs) [24]. In addition to its ligands, recent studies demonstrated that post-translational modifications (PTMs) of PPARγ, including phosphorylation, small ubiquitin-like modifier (SUMO)-ylation, acetylation, and ubiquitination, are major regulatory mechanisms of PPARγ activation and PPARγ-mediated gene transcription [18,25,26]. Of these, phosphorylation is thought to be a crucial regulator of the transcriptional regulatory activity of PPARγ. Phosphorylation of PPARγ at Ser112 (pSer112) by mitogen-activated protein kinases (MAPKs) suppresses its transcriptional activity, and therefore adipocyte differentiation [27]. Recently, we demonstrated that PPARγ is phosphorylated at Ser273 by cyclin-dependent kinase 5 (CDK5)/extracellular signal-regulated kinase (ERK), and this phosphorylation does not affect its adipogenic activity but instead dysregulates a specific set of genes, the expression of which is altered in obesity and/or type 2 diabetes [28,29].

Although the phosphorylations of PPARγ are known to be performed by CDK5 and MAPKs, little is known about the intrinsic negative regulators of such phosphorylations. It was reported that protein phosphatase 5 specifically dephosphorylates PPARγ and glucocorticoid receptor (GR) [30]. In addition, protein phosphatase Mg^2+^/Mn^2+^-dependent 1B (PPM1B), and protein phosphatase 2C delta (WIP1), which are serine/threonine phosphatases belonging to the protein phosphatase Mg^2+^/Mn^2^ (PPM) family, dephosphorylate PPARγ at Ser112, which activates its transcriptional activity, thereby increasing adipogenesis and fat accumulation in vitro and in vivo [31,32]. However, the phosphatases responsible for the dephosphorylation of PPARγ at Ser273 are still unknown. Since it was shown that the members of PPM family phosphatase function as a PPARγ phosphatase [31,32], we hypothesized that PPM family phosphatases could dephosphorylate PPARγ at Ser273, and we interrogated seven members of the PPM family using a PPARγ dephosphorylation assay. Among them, we identified a novel protein phosphatase Mg^2+^/Mn^2+^-dependent 1A (PPM1A), which specifically and directly dephosphorylates PPARγ at Ser273.

It was reported that the activation of PPM1A requires magnesium and/or manganese ions [33,34]. PPM1A specifically binds to its substrates and catalyzes the dephosphorylation of these substrates [35,36]. For example, p38 MAPK, small mothers against decapentaplegic homolog 2 (SMAD2) and SMAD3, MAPK kinase 4 (MKK4), and MKK6 are reported as specific substrates of PPM1A, and this direct interaction is involved in the regulation of diverse cellular processes, including apoptosis, the cell cycle, and differentiation [37,38]. In this study, we show that PPM1A-mediated dephosphorylation of PPARγ at Ser273 in mature adipocytes restores the expression of most of the genes that are dysregulated by pSer273. These data strongly suggest that PPM1A plays an important role in the regulation of the diabetic gene reprogramming by dephosphorylating PPARγ at Ser273, and that PPM1A may represent a therapeutic target for obesity and type 2 diabetes.

## 2. Materials and Methods

### 2.1. Cell Culture

3T3-L1, human embryonic kidney 293 (HEK-293) cells were purchased from American Type Culture Collection (ATCC, Manassas, VA, USA) and cultured in Dulbecco’s modified Eagle’s medium (DMEM) containing 10% fetal bovine serum (Gemini Bio-products, West Sacramento, CA, USA) and 1% penicillin/streptomycin (Thermo Fisher Scientific, Waltham, MA, USA) at 37 °C under 5% CO_2_ conditions. Adipocyte differentiation was performed by a previously described procedure [28]. Briefly, 3T3-L1 pre-adipocytes were cultured with DMEM containing 10% fetal bovine serum (FBS) until they reached 100% confluence. After 2 days, the maintaining media was changed by differentiation media containing 3-isobutyl-1-methylxanthine (IBMX, 0.5 mM), dexamethasone (1 μM), and insulin (850 nM). Then, the media was changed by differentiation media containing insulin (850 nM) once every 2 days. All experiments performed 7 days after changing the differentiation media. All chemicals used for cell culture were purchased from Sigma-Aldrich (St. Louis, MO, USA).

### 2.2. Expression Plasmids and RNA Interference

Expression plasmids for FLAG epitope-tagged wild-type (WT) and PPARγ^S273A^ were used as described previously [28]. WT human PPM1A, 1B, 1D, 1F, 1J, 1K, and 1M were obtained from Korea Research Institute of Bioscience and Biotechnology (KRIBB). PPM1A, R174G, or D239N substitutions were constructed using QuickChange II Site-Directed Mutagenesis Kit obtained from Agilent Technologies (Santa Clara, CA, USA). All mammalian expression plasmids were transfected using Lipofectamine^TM^ 2000 transfection reagent (ThermoFisher Scientific, Waltham, MA, USA) following the manufacturer’s instructions. siRNAs for mouse PPM1A #1 (5′-CCAAGTGGACTTGAG-ACATGGTCAT-3′) and #2 (5′-GACATGGTCATTCTTTGCTGTATAT-3′) were purchased from Shanghai GenePharma (Shanghai, China). 3T3-L1 cells were transfected with siRNAs using Lipofectamine^TM^ RNAiMax transfection reagent (ThermoFisher Scientific, Waltham, MA, USA).

### 2.3. Lentivirus Production and Infection

Murine PPM1A WT, R174G, and D239N were sub-cloned into pLenti-GIII-CMV-GFP-2A-Puro (Applied Biological Materials, Richmond, BC, Canada). For lentiviral package plasmids, we used pMDLg/pRRE and pRSV/REV, while pLV-VSVG was used as an envelope plasmid (Addgene, #12251, #12253, #82724). HEK-293T cells were transfected with each lentiviral vector, packaging plasmids, and envelope plasmid under 5:2:2:1 ratio. Produced lentivirus from media were infected into 3T3-L1 adipocytes with polybrene (Sigma-Aldrich, St. Louis, MO, USA).

### 2.4. Immunoprecipitation and Immunoblotting

HEK-293 cells expressing PPARγ were treated with 500 nM of phorbol 12-myristate 13-acetate (PMA), and total cell lysates were incubated with FLAG M2 agarose (Sigma-Aldrich, St. Louis, MO, USA) at 4 °C. Immunoprecipitates, total cell, or tissue lysates were analyzed with phospho-specific antibody against PPARγ at Ser273 [28], anti-PPARγ at Ser112 (Biocompare, San Francisco, CA, USA), or anti-PPARγ antibody (Santa Cruz Biotechnology, Dallas, TX, USA). The antibodies used in this study including anti-glutathione *S*-transferase (GST), aP2, and C/EBPα were purchased from Santa Cruz Biotechnology (Dallas, TX, USA), while anti-HA, p-ERK1/2, ERK, adiponectin, β-tubulin, Histone 2A, and HSP90 were purchased from Cell Signaling Technology (Danvers, MA, USA).

### 2.5. In Vitro Phosphatase Assay

PPM1A human recombinant and active ERK1 were purchased from Prospec (Ness-Ziona, Israel) and SignalChem (Richmond, British Columbia, Canada), respectively. Glutathione *S*-transferase (GST)-fused PPARγ was incubated with active ERK1 in kinase assay buffer (25 mmol/L Tris-HCl, pH 7.5, 5 mmol/L β-glycerophosphate, 2 mmol/L DTT, 0.1mmol/L Na_3_VO_4_, 10 mmol/L MgCl_2_) containing 20 μmol/L ATP for 20 min at 30 °C. Then, purified phosphorylated GST-fused PPARγ was added into PPM1A human recombinant containing PPM1A phosphatase buffer [38] and incubated 30 min at 30 °C. To check the effect of catalytic inactive mutants of PPM1A (R174G and D239N) in dephosphorylation of PPARγ, HEK-293 cells were transfected with mutants expressing vector. Immunopurified PPM1As were incubated with phosphorylated GST-fused PPARγ in PPM1A phosphatase buffer for 30 min at 30 °C. All reactions were terminated by the addition of protein sample buffer. Phosphorylation of PPARγ was analyzed by SDS-PAGE with phospho-specific antibody against PPARγ at Ser273, anti-GST, or PPM1A antibodies.

### 2.6. Dot Blot Assay

PPM1A human recombinant was incubated with phospho-Ser273 peptide, which conserves the amino acid sequence of PPARγ (TGKTTDK-pS-PFVIYDM, New England peptide, Gardner, MA, USA) in PPM1A phosphatase assay buffer for 20 min at 30 °C. A portion was dotted on nitrocellulose membrane and dried for 3 h at real-time (RT). After dry step, the membrane was washed by Tris-buffered saline and Tween 20 (TBST) 3 times per 10 min. Washed membrane was blocked by 5% bovine serum albumin (BSA) dissolved in TBST and immunoblotting was performed with phospho-specific antibody against PPARγ at Ser273 as described previously.

### 2.7. Subcellular Fractionation

Fully differentiated 3T3-L1 cells were washed by ice-cold PBS buffer, and the centrifuged pellet was resuspended with 20 mM hydroxyethyl piperazine ethane sulfonicacid (HEPES) pH 7.4. Using a 1 mL syringe, cells were lysed 10 times by needle suspension. After incubating for 20 min on ice, lysates were fractionated into nuclei in pellet and cytoplasm in supernatant by 720× *g* centrifugation for 5 min at 4 °C. Then the pellet was resuspended with 5 mM HEPES pH 7.9 added with 400 mM NaCl and needle suspension was performed 20 times. After 30 min on ice, nuclear proteins acquired by centrifugation at 24,000× *g* for 30 min at 4 °C. Cytoplasmic and nuclear proteins were quantified by Bradford assay and proteins were detected by Western blotting.

### 2.8. In Vitro Insulin-Resistance (IR) Model

For an experimental procedure of IR-induced model in vitro, we followed Lo. et. al. [39]. Briefly, fully differentiated 3T3-L1 cells were washed by PBS buffer, and changed to low-glucose (1 g/L) DMEM containing 0.5% BSA and 2.5 nM of TNF-α, without serum. After 24 h, total RNAs were isolated.

### 2.9. Real-Time RT-PCR (Quantitative PCR, qPCR)

Total RNAs were isolated using TRIzol reagent purchased from Thermo Fisher Scientific (Waltham, MA, USA). Reverse-transcription of the RNA was performed with ABI Reverse Transcription Kit. qPCR was performed using 7900HT Fast Real-Time PCR System (Life Technologies, Carlsbad, CA, USA) following the manufacturer’s instructions. Relative mRNA expression levels of each gene were normalized to 36B4 or TATA-binding protein TBP. Each analysis was conducted in triplicates. Data were analyzed by Microsoft Excel (Microsoft, Redmond, WA, USA).

### 2.10. Correlation Analysis in Human Adipose Tissues

To analyze the correlation between PPM1A and PPARγ phosphorylation at Ser273 in lean and obese humans, GSE55200, from public Gene Expression Omnibus (GEO) database accessible at the National Center for Biotechnology Information (NCBI, Bethesda, MD, USA), was analyzed. We analyzed the expression levels of PPM1A and genes responsive to PPARγ phosphorylation at Ser273 as described previously in human subcutaneous adipose tissue [40,41].

### 2.11. Animals

All animal experiments were performed according to procedures approved by the Ulsan National Institute of Science and Technology’s Institutional Animal Care and Use Committee. Five-week-old male C57BL/6J mice (DBL, Chungbuk, Korea) were fed a high-fat diet (60% kcal fat, D12492, Research Diets Inc., New Brunswick, NJ, USA) for 8 weeks. The *db/db* mice used in this study were purchased from the Jackson Laboratory (Bar Harbor, ME, USA).

## 3. Results

### 3.1. PPM1A is a Phosphatase that Acts on PPARγ

**Figure 1:**
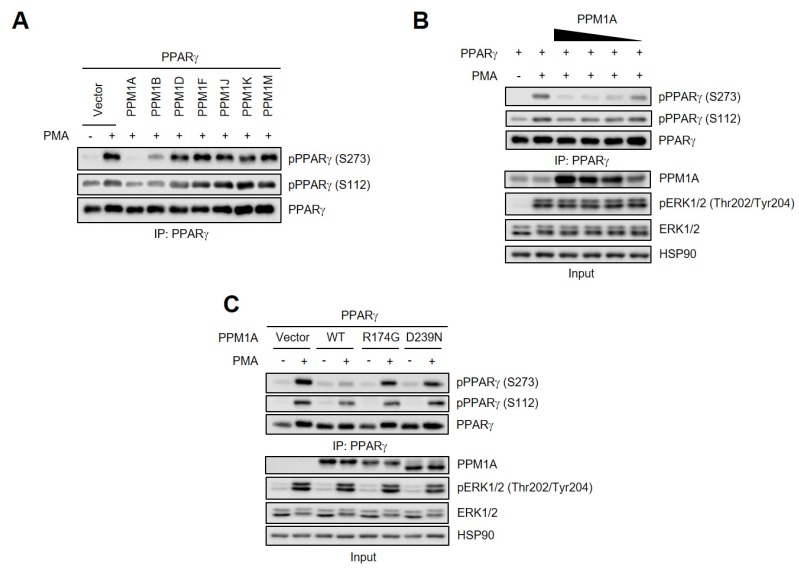
Identification of novel phosphatase of peroxisome proliferator-activated receptor (PPARγ) at Ser273 and Ser112. (**A**) Human embryonic kidney 293 (HEK-293) cells were transfected with protein phosphatase Mg^2+^/Mn^2^ (PPM) and PPARγ. PPARγ was phosphorylated by phorbol myristate acetate (PMA) (500 nM) for 30 min. Immunoprecipitated PPARγ were analyzed by Western blotting. (**B**) HEK-293 cells were transfected with protein phosphatase Mg^2+^/Mn^2+^-dependent 1A (PPM1A) with PPARγ in a dose-dependent manner. PPARγ phosphorylation was analyzed in immunoprecipitated cell lysates, and PPM1A, extracellular signal-regulated kinase (ERK)1/2, and ERK1/2 phosphorylation were measured in whole cell lysate (input) by Western blotting. (**C**) Wild-type (WT) PPM1A and its catalytically inactive mutants (R174G and D239N) were transfected into HEK-293 cells with PPARγ. PPARγ phosphorylation was analyzed by Western blotting. HSP90 was used as loading control.

Recent studies showed that members of the PPM family could be the serine phosphatases of PPARγ [31,32]. Therefore, we aimed to identify the phosphatase for the Ser273 residue of PPARγ by interrogating a number of PPMs. Of the seven tested, we found that both PPM1A and PPM1B specifically dephosphorylated PPARγ at Ser273 after phorbol myristate acetate (PMA) treatment, and that PPM1A did so more effectively than PPM1B (Figure 1A). Consistent with previous reports, PPM1B dephosphorylates PPARγ at Ser112 [31], but interestingly, PPM1A also inhibits PMA-mediated PPARγ phosphorylation at Ser112. To further investigate the effects of PPM1A on the phosphorylation of PPARγ, we overexpressed PPM1A in HEK-293 cells and found that the degree of dephosphorylation of PPARγ by PPM1A is proportional to the level of PPM1A expression (Figure 1B). It was reported that the phosphorylation of PPARγ at Ser273 is mediated by ERK [29]. Therefore, we tested whether the dephosphorylation by PPM1A might be the result of an inhibition of ERK activation following PMA treatment. However, forced expression of PPM1A did not change the degree of PMA-induced ERK phosphorylation, suggesting that the dephosphorylation of PPARγ by PPM1A is not caused by the indirect inhibition of ERK.

To determine whether PPM1A phosphatase activity is required for PPARγ dephosphorylation, we generated two catalytically inactive PPM1A mutants, R174G and D239N [34], and found that both lost their ability to dephosphorylate PPARγ at Ser112 and Ser273, while not affecting PMA-induced ERK activation (Figure 1C). Taken together, these results strongly suggest that PPM1A is a specific phosphatase of PPARγ at both Ser112 and Ser273 in the cell culture system.

### 3.2. PPM1A Directly Dephosphorylates PPARγ at Ser273 but not Ser112

**Figure 2:**
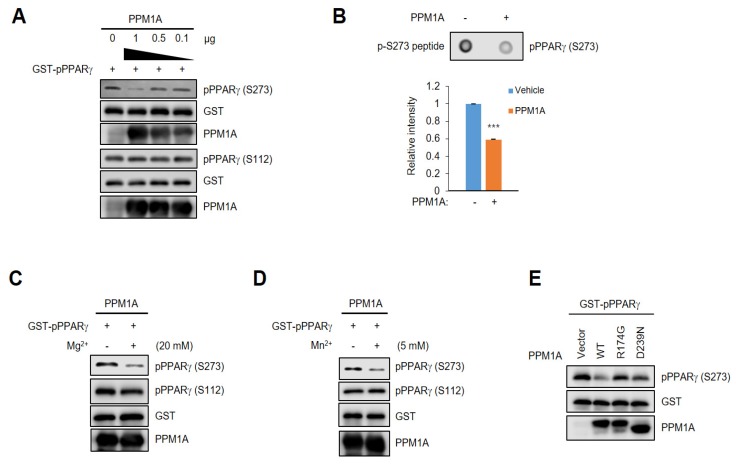
PPM1A directly dephosphorylates PPARγ at Ser273. (**A**) In vitro PPM1A phosphatase assay was performed with full-length glutathione *S*-transferase (GST)-fused phosphorylated PPARγ and recombinant PPM1A. Total PPARγ and PPARγ phosphorylation were analyzed by Western blotting. (**B**) Dot blot assay was performed using phospho-peptide flanking pSer273 residue with recombinant PPM1A. S273 phosphorylation of PPARγ was analyzed by Western blotting. Three independent experiments were performed and the level of PPARγ phosphorylation was quantified by Image Studio. (**C**,**D**) In vitro PPM1A phosphatase assay was performed in the presence of magnesium and manganese ions (20 mM and 5 mM, respectively). Western blots were performed to detect each protein indicated. (**E**) In vitro PPM1A phosphatase assay was performed with GST-fused phosphorylated PPARγ and immune-purified WT PPM1A and two mutants. PPARγ phosphorylation was analyzed by Western blotting. *** *p* < 0.001; Error bars indicate standard error of the mean (SEM).

We next determined the effect of PPM1A on PPARγ dephosphorylation in a cell-free system. After purifying a glutathione S-transferase (GST)-PPARγ recombinant fusion protein, we directly phosphorylated PPARγ at Ser273 using active ERK. Then, we performed an in vitro phosphatase assay using recombinant active PPM1A. As shown in Figure 2A, PPM1A directly dephosphorylated PPARγ at Ser273 but not at Ser112. These results indicate that PPM1A is a direct phosphatase of PPARγ at Ser273, and that the dephosphorylation by PPM1A at Ser112 in cells is indirect. Furthermore, when we incubated a phosphopeptide including the pSer273 residue with active PPM1A, phosphorylation at Ser273 significantly decreased (Figure 2B). Due to PPM1A [33,34], we further tested the effect of both ions on dephosphorylating PPARγ at Ser273. As shown in Figure 2C,D, the phosphatase activity of PPM1A against pSer273 is much higher in the presence of both Mg^2+^ and Mn^2+^ ions, but pSer112 is unchanged. Next, to determine whether the catalytic activity of PPM1A is required for the dephosphorylation of PPARγ, we immunopurified wild-type (WT) PPM1A and the two catalytic inactive mutants of PPM1A from HEK-293 cells. After phosphorylating PPARγ at Ser273, we incubated phosphorylated PPARγ with PPM1As. WT PPM1A, but not the two catalytically inactive mutants, dephosphorylated PPARγ (Figure 2E). Taken together, these results strongly suggest that PPM1A is a specific and direct phosphatase of PPARγ at Ser273, but not Ser112.

### 3.3. PPM1A Physically Interacts with PPARγ

**Figure 3:**
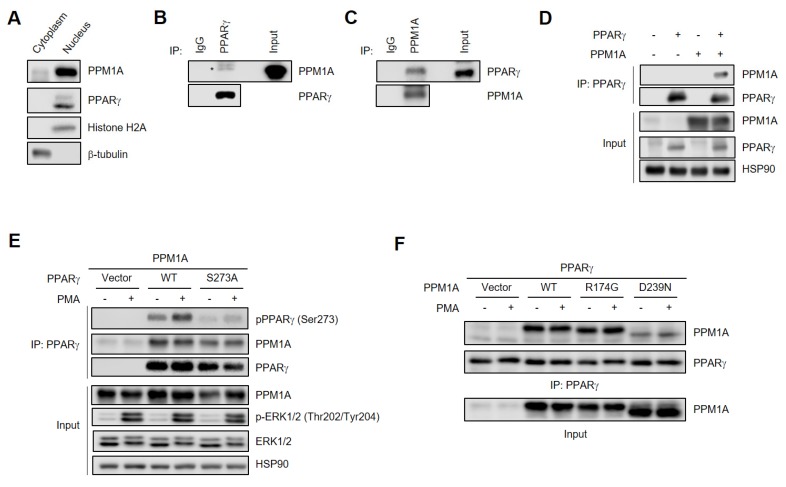
PPM1A directly interacts with PPARγ in adipocyte. (**A**) Western blot analysis of subcellular fractions in 3T3-L1 adipocytes. Histone H2A and β-tubulin was detected for nucleus and cytoplasmic marker protein, respectively. (**B, C**) Endogenous PPM1A and PPARγ were immunoprecipitated and physical interaction between them was analyzed by Western blotting. (**D**–**F**) PPARγ was phosphorylated by PMA (500 nM) for 30 min in HEK-293 cells. (D) HEK-293 cells were transfected with PPARγ and PPM1A. Immunoprecipitation was performed, and each protein was analyzed by Western blotting. (**E**) WT PPARγ and phospho-defective mutant of PPARγ (S273A) were transfected in HEK-293 cells with PPM1A. Immunoprecipitation was performed, and each protein was analyzed by Western blotting. (**F**) WT PPM1A and its catalytically inactive mutants R174G and D239N were transfected in HEK-293 cells with PPARγ. Immunoprecipitation was performed and each protein was analyzed by Western blotting.

Since PPM1A directly dephosphorylates PPARγ, it is expected that they would be localized to the nucleus. Thus, we analyzed the subcellular localization of both PPM1A and PPARγ and found that both were predominantly localized in the nucleus (Figure 3A). Next, we performed co-immunoprecipitation experiments to determine whether PPM1A binds to PPARγ. Endogenous PPM1A specifically interacts with endogenous PPARγ in adipocytes and vice versa (Figure 3B,C). In addition, PPM1A specifically interacts with PPARγ when co-expressed in HEK-293 cells (Figure 3D). Interestingly, a phosphorylation-defective mutant of PPARγ (S273A) interacts normally with PPM1A. Furthermore, PMA does not affect the interaction between PPM1A and PPARγ, suggesting that the phosphorylation status of PPARγ is not critical for its interaction with PPM1A. The catalytically inactive PPM1A mutant (R174G) retains its ability to interact with PPARγ, but the D239N mutation significantly reduces the interaction (Figure 3F). These results are consistent with previous reports that Asp239 residue is critical for substrate binding by coordinating to metal M2 and bridging with metal-bound water [42]. Together, these results indicate that PPM1A physically interacts with PPARγ in a phosphorylation-independent manner.

### 3.4. PPM1A Specifically Regulates the Expression of a Diabetic Gene Set that is Dysregulated by pSer273

**Figure 4:**
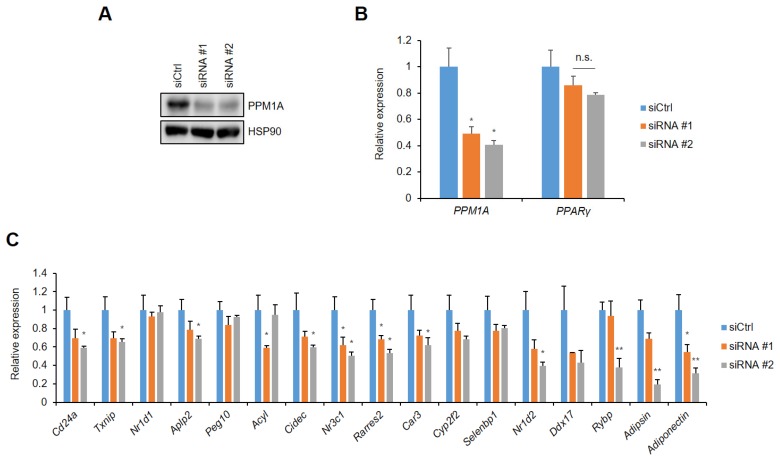
Knockdown of PPM1A downregulates gene sets dysregulated by pSer273. 3T3-L1 adipocytes were transfected with specific human siRNAs targeting PPM1A. (**A**) Western blot of 3T3-L1 cell lysates transfected by siRNAs. (**B, C**) 3T3-L1 cells were introduced with specific mouse PPM1A targeting siRNAs. Relative mRNA expression of PPM1A and PPARγ are shown in (**B**), and expression of specific gene sets dysregulated by pS273 are shown in (**C**). * *p* < 0.05; ** *p* < 0.01; Error bars indicate SEM; n.s., not significant.

To elucidate the functional roles of PPM1A on pSer273, we investigated the effect of PPM1A on pSer273-mediated gene regulation in adipocytes. Specific siRNAs targeting PPM1A were introduced into adipocytes, which dramatically reduce the expression of PPM1A (Figure 4A). The expression of PPARγ is not changed by PPM1A knockdown (Figure 4B). Importantly, most of the genes known to be specifically dysregulated by pSer273 are sensitive to the knockdown of PPM1A (Figure 4C). These include adiponectin and adipsin, which are adipokines that are important regulators of insulin sensitivity and glucose homeostasis [14,15,16,17].

**Figure 5:**
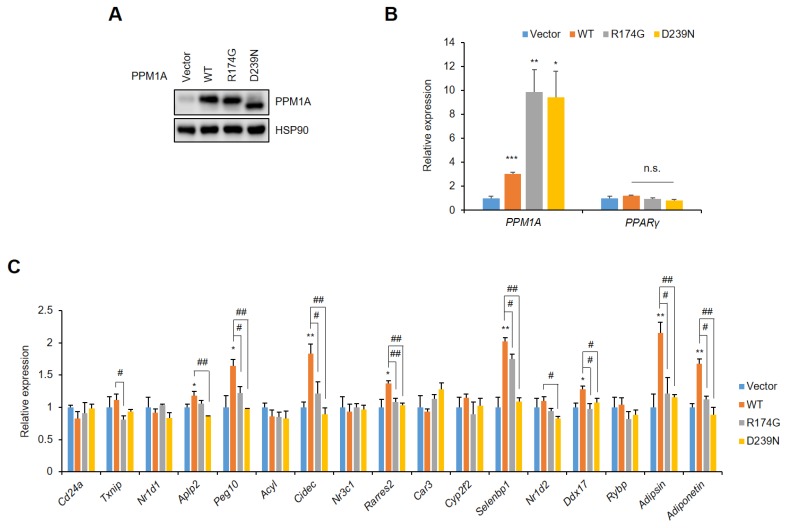
Forced expression of PPM1A restores the diabetic gene set in adipocytes. 3T3-L1 adipocytes were infected with lentivirus expressing of WT PPM1A and its mutants (R174G and D239N). (**A**) Western blot of 3T3-L1 cell lysates transfected by PPM1A. (**B, C**) 3T3-L1 cells expressing WT PPM1A and its mutants were differentiated into adipocytes. Relative mRNA expression of PPM1A and PPARγ is shown in (B), and expression of diabetic gene set regulated by pSer273 is shown in (C). * *p* < 0.05; ** *p* < 0.01; *** *p* < 0.001 vs. ‘vector’; # *p* < 0.05; ## *p* < 0.01 vs. ‘WT’; Error bars indicate SEM; n.s., not significant.

In contrast, the forced expression of PPM1A in adipocytes has opposite effects to the knockdown of PPM1A with gene regulation. The lentiviral overexpression of WT PPM1A and two catalytic inactive mutants does not change the expression of PPARγ in adipocytes (Figure 5A,B). However, WT PPM1A markedly increases the expression of most of the specific gene set that is dysregulated by pSer273, but the R174G and D239N mutants do not (Figure 5C). Taken together, these results strongly suggest that PPM1A regulates the diabetic gene program by dephosphorylating PPARγ at Ser273.

### 3.5. Knockdown of PPM1A Devastates Insulin Resistant Status in Adipocytes

**Figure 6:**
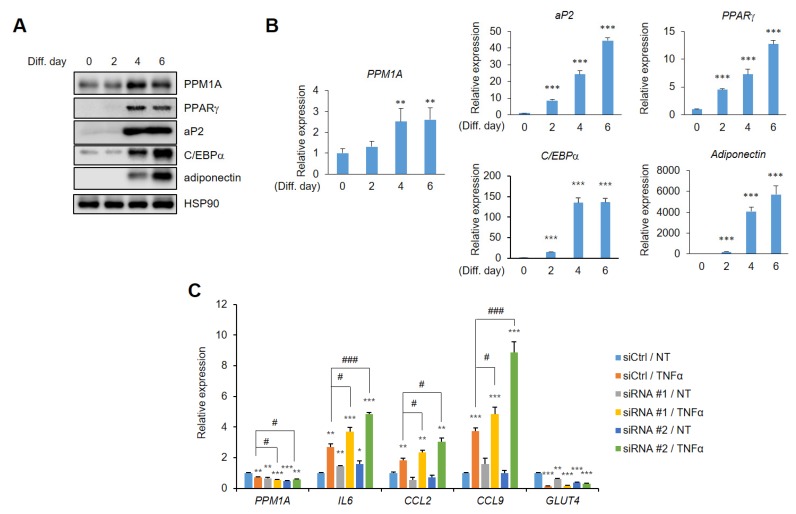
PPM1A ameliorates insulin resistance in adipocytes. (**A**) Western blot of PPM1A and adipogenic marker proteins from 3T3-L1 cells during adipogenesis. (**B**) Relative mRNA expression of PPM1A and adipogenic marker genes were investigated by RT-qPCR (*n* = 3). (**C**) Control and PPM1A siRNAs introducing 3T3-L1 adipocytes were treated with tumor necrosis factor-α (TNF-α) for 24 h and mRNA expression of PPM1A, insulin resistance marker genes (*IL6*, *CCL2*, *CCL9*), and insulin sensitivity marker gene (*GLUT4*) was measured by RT-qPCR. * *p* < 0.05; ** *p* < 0.01; *** *p* < 0.001 vs. ‘siCtrl/NT’; # *p* < 0.05; ### *p* < 0.001 vs. ‘siCtrl/TNF-α’; Error bars indicate SEM.

Next, we aimed to determine the physiological relevance of PPM1A in adipocyte biology. Western blotting and real-time PCR analysis revealed that PPM1A is expressed in pre-adipocytes, and that its expression significantly increases during adipogenesis (Figure 6A). In addition, the expression of PPARγ and adipogenic marker genes, including aP2, C/EBPα, and adiponectin, also significantly increases (Figure 6B), indicating that PPM1A might have important roles in differentiated adipocytes. It is well characterized that pS273 is linked to obesity and insulin resistance [28,29] and blocking pSer273 by specific PPARγ ligands shows improved insulin sensitivity [43,44,45].

Thus, we tried to determine whether PPM1A has a potential role in insulin sensitivity. It was shown that insulin resistance can be modeled by treating fully differentiated adipocytes with TNF-α [39]. Therefore, we treated adipocytes with TNF-α, which dramatically increased the expression of genes involved in insulin resistance in adipocytes, such as IL-6, CCL2, and chemokine ligand 9 (CCL9), but significantly reduced glucose transporter 4 (GLUT4) expression (Figure 6C). Interestingly, the expression of PPM1A was significantly decreased in this model, and those genes were dramatically altered by PPM1A knockdown in adipocytes. The expression of IL-6, CCL2, and CCL9 dramatically increases by specific knockdown of PPM1A and the expression of GLUT4 is significantly decreased by PPM1A knockdown. These results strongly suggest that PPM1A is positively associated with insulin sensitivity in adipocytes.

### 3.6. PPM1A is Negatively Correlated with pSer273

**Figure 7:**
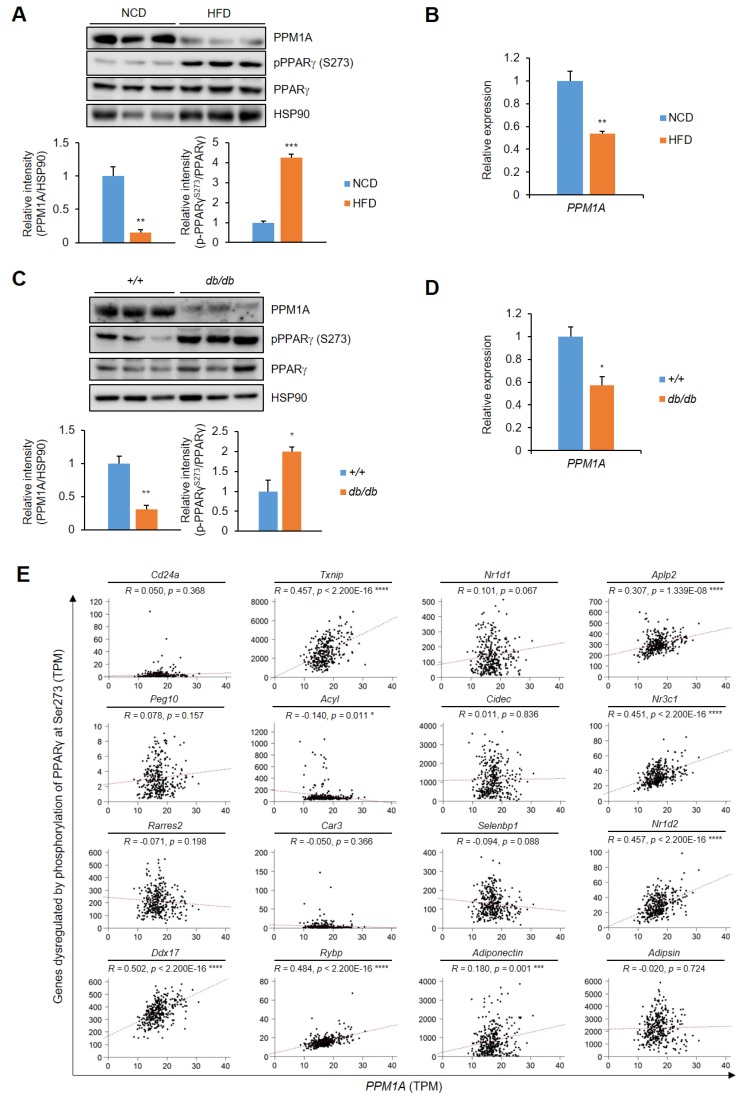
Negative correlation between PPM1A and the phosphorylation of PPARγ at Ser273 in pathophysiological conditions both in mice and humans. (**A**) Western blot analysis of PPM1A, PPARγ, and PPARγ phosphorylation at Ser273 in adipose tissue of both normal chow diet (NCD)-fed mice and high-fat diet (HFD)-fed mice for 8 weeks. The expression of PPM1A and the phosphorylation of PPARγ at Ser273 are normalized by HSP90 and total PPARγ, respectively (shown as bar graphs). (**B**) Relative mRNA expression of PPM1A in adipose tissue in the same mice (*n*=3). (**C**) Western blot of PPM1A, PPARγ, and PPARγ phosphorylation at Ser273 in adipose tissue of 10 week-old C57BL6/J and *db/db* mice. The expression of PPM1A and the phosphorylation of PPARγ at Ser273 are normalized by HSP90 and total PPARγ, respectively (shown as bar graphs). (**D**) Relative mRNA expression of PPM1A in adipose tissue in the same mice (*n* = 3). (**E**) Pearson correlation coefficient between PPM1A and gene set responsive to pSer273 within human subcutaneous adipose tissue is calculated. * *p* < 0.05; ** *p* < 0.01; *** *p* < 0.001; **** *p*< 0.0001; Error bars indicate SEM.

We then investigated the physiological relevance of PPM1A in adipose tissue. First, we used two different models: high-fat diet (HFD)-induced obese mice and genetically obesity mice (*db/db*). As shown in Figure 7A,B, the phosphorylation of PPARγ at Ser273 is very high in the adipose of these mice, as previously reported [28]. Interestingly, PPM1A expression was significantly lower in the adipose tissue of both HFD-fed and *db/db* mice than in control mice, indicating that there is a negative association between PPM1A expression and pSer273 (Figure 7C,D). To further evaluate this correlation, we interrogated a public GSE55200 database which compares gene expression in lean and obese human subcutaneous adipose tissue (Table 1). The expression of PPM1A is significantly lower in obese than in normal adipose. By contrast, the expression of pro-inflammatory cytokines, such as TNF-α and CCL2, is much higher. Furthermore, the expression of adiponectin and GLUT4 is significantly lower. In addition, most of the genes dysregulated by pSer273 are significantly increased in obese adipose tissue (Table 1). To further check the correlation between PPM1A expression and phosphorylation of PPARγ at Ser273, we analyzed the relative expression of PPM1A and pSer273 specific genes in human subcutaneous adipose tissue using GTEx database (Figure 7E). Indeed, the expression of most of the genes dysregulated by pSer273 positively correlated with the expression of PPM1A. Taken together, our results strongly suggest that PPM1A is negatively correlated with pSer273 in both mice and humans.

## 4. Discussion

**Figure 8:**
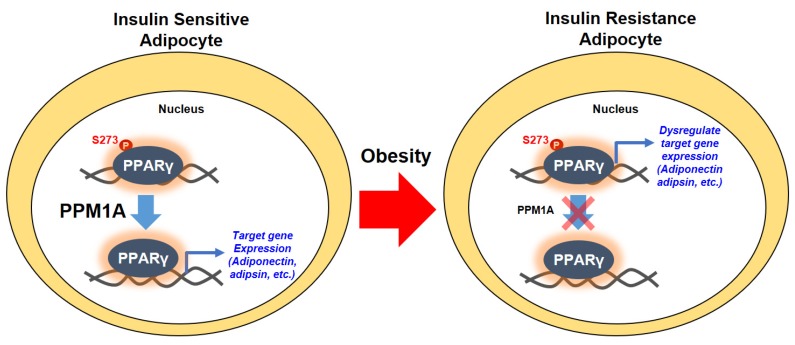
Schematic model of dephosphorylation of PPARγ at Ser273 by PPM1A. In normal status (insulin sensitive condition), a degree of PPARγ phosphorylation at Ser273 is reduced by the action of PPM1A in adipocytes. Dephosphorylated PPARγ orchestrates the specific target gene expression including insulin-sensitizing adipokines, such as adiponectin and adipsin. In obese status (insulin resistant condition), however, phosphorylation of PPARγ at Ser273 significantly increases because of the lower level of PPM1A expression. Phosphorylated PPARγ dysregulates the diabetic gene programming so that adipocytes change into insulin resistance phenotype.

Post-translational modifications of PPARγ orchestrate various functions of the protein [18,25,26]. In obesity, phosphorylation of PPARγ on the Ser273 residue by CDK5 leads to dysregulation of a specific gene set [28]. In addition to the effect of CDK5 on Ser273, ERK is shown to regulate the diabetogenic effect of PPARγ [29]. These studies collectively suggest that pSer273 is a potential therapeutic target for metabolic diseases. In this study, we demonstrate that PPM1A is a novel protein phosphatase of PPARγ that directly dephosphorylates pSer273, reprogramming anti-diabetic gene expression (Figure 8). First, PPM1A interacts with PPARγ and directly dephosphorylates at Ser273. Second, genetically altered expression of PPM1A significantly affects the expression of the gene set that is dysregulated by pSer273 in adipocytes. Third, PPM1A expression negatively correlates with the degree of phosphorylation of PPARγ, both in obese mice and human adipose tissue. Finally, transcriptomic analyses are highly consistent with this relationship.

Although a number of studies identified phosphatases of PPARγ, most focused on pSer112. Our results show that PPM1A reduces the level of phosphorylation at Ser112, but an in vitro phosphatase assay indicates that this dephosphorylation is not direct. The Ser112 residue of PPARγ is phosphorylated by ERK and c-Jun N-terminal kinase (JNK), but not p38 MAPK, and reduces PPARγ activity. Paradoxically, the same site can be phosphorylated by CDK7 and CDK9, which increases the activity of PPARγ [46,47]. We showed that there is no effect of PPM1A on the phosphorylation of ERK in cells (Figure 1), but the exact mechanism whereby PPM1A reduces pSer112 in adipocytes remains to be elucidated. Further mechanistic studies should be conducted to confirm the direct target of PPM1A that affects phosphorylation at Ser112.

Recent studies have shown that PPM1A plays an important role in metabolism [37,48,49,50]. Unhealthy adipocytes are exposed to adipose tissue-resident macrophages, which are phenotypically defined as M1 macrophages [2,51,52,53,54]. Pro-inflammatory cytokines, including IL-1β, IL6, and TNF-α, secreted by M1-polarized macrophages, alter the metabolic phenotype of adipose tissue [55,56,57,58]. Smith et al. showed that PPM1A regulates monocyte–macrophage differentiation, reduces the expression of M1 macrophage marker genes, and inhibits the production of pro-inflammatory cytokines [59]. In addition, PPM1A terminates TNF-α-induced inhibitor κB kinase beta (IKKβ) activation and transforming growth factor beta (TGFβ) signaling by dephosphorylating IKKβ and SMAD2/3, respectively [38,60]. These results suggest that PPM1A plays an important role in inhibiting pro-inflammatory cytokine-induced inflammatory signaling. Interestingly, our previous studies demonstrated that dephosphorylation of PPARγ at Ser273 is responsible for anti-inflammatory functions by controlling macrophage polarization which decreased M1 polarization, but increased M2 phenotypes [44,45]. Furthermore, we found that TNF-α-induced phosphorylation of PPARγ at Ser273 increased pro-inflammatory response in adipocytes [45]. Notably, insulin-resistant TNF-α-treated differentiated 3T3-L1 adipocytes show significantly lower PPM1A expression. Specifically, PPM1A recovers insulin sensitivity-related gene expression which is dysregulated by TNF-α (Figure 6) and upregulates the expression of insulin-sensitizing adipokines, adiponectin, and adipsin (Figure 4 and Figure 5). Together, we highlight that PPM1A may represent a therapeutic target for metabolic diseases in many aspects, such as increasing anti-inflammatory responses and enhancing the secretion of insulin-sensitizing adipokines which depends on dephosphorylation of PPARγ at Ser273.

Both extracellular and intracellular magnesium deficits are frequently detected in patients with type 2 diabetes and in overweight or obese children [61,62]. Low Mg^2+^ concentrations are closely associated with post-receptor impairment in the insulin signaling pathway [61,63,64]. In addition, a series of recent studies revealed lower concentrations of Mg^2+^ in the liver, small intestine, and bone of lean mice compared with obese mice [65,66,67]. Furthermore, epidemiological studies conducted worldwide suggest that a higher intake of Mn^2+^ is associated with a lower risk of obesity [61,68,69]. Thus, the Mg^2+^/Mn^2+^-dependency of PPM1A for the dephosphorylation of PPARγ is an important consideration when evaluating the phosphatase as a therapeutic target. However, the intracellular concentrations of Mg^2+^ and Mn^2+^ are strictly regulated, and failure of homeostasis is involved in several diseases [70,71]. For example, hypermagnesemia is common in renal failure patients [72], and an accumulation of manganese in the brain can cause neurodegenerative disorders [73]. Thus, further studies are required to uncover the molecular mechanisms which promote the expression of PPM1A and to develop the specific activators of PPM1A rather than regulating the intracellular concentrations of Mg^2+^ and Mn^2+^.

Our data suggest the critical roles for PPM1A as a novel phosphatase of PPARγ, which finely orchestrates diabetic gene expression in adipocytes. Although further studies are required to elucidate the relationship between PPM1A and metabolic diseases, our study proposes that PPM1A may represent a promising therapeutic target for obesity and metabolic disorders.

## Figures and Tables

**Table 1:** Differential gene expression in subcutaneous adipose tissue of unhealthy obese individuals.

Affymetrix ID	Gene Symbol	Gene Name	log_2_ FC (vs. Lean Healthy)	*p*-Value
16784909	Ppm1a	Protein phosphatase, Mg^2+^/Mn^2+^ dependent, 1A	–0.02583	0.004725 **
17117110	Cd24a	CD24 molecule, type A	–0.03623	0.291983
16669796	Txnip	Thioredoxin interacting protein	–0.02935	0.005334 **
16844294	Nr1d1	Nuclear receptor subfamily 1 group D member 1	0.054739	0.042975 *
16733435	Aplp2	Amyloid beta precursor like protein 2	0.012981	0.048507 *
17048563	Peg10	Paternally expressed 10	0.158202	0.018165 *
16852660	Acyl	ATP citrate lyase	−0.10524	0.037731 *
16950609	Cidec	Cell death inducing DFFA like effector C	−0.04005	0.000383 ***
17001100	Nr3c1	Nuclear receptor subfamily 3 group C member 1	−0.07386	0.000149 ***
17064251	Rarres2	Retinoic acid receptor responder 2	−0.08898	0.011576 *
17070441	Car3	Carbonic anhydrase 3	−0.394	0.001141 **
16693082	Selenbp1	Selenium binding protein 1	−0.04191	0.034659 *
16938378	Nr1d2	Nuclear receptor subfamily 1 group D member 2	−0.02497	0.070348
16935042	Ddx17	DEAD-box helicase 17	−0.00854	0.177737
16956244	Rybp	RING1 And YY1 binding protein	0.002819	0.427001
16949397	Adipoq	Adiponectin, C1Q annd collagen domain containing	−0.02938	0.000331 ***
16856299	Cfd	Complement factor D, adipsin	0.00749	0.227859
16830343	Slc2a4	Solute carrier family 2 member 4, GLUT4	−0.46174	1.4 × 10^−7^ ***
17001763	Tnf	Tumor necrosis factor	0.055804	2.1 × 10^−5^ ****
16833204	Ccl2	C-C motif chemokine ligand 2	0.364464	4.52 × 10^−5^ ****

Expression of PPM1A, pSer273 responsive genes (*Cd24a*, *Txnip*, *Nr1d1*, *Aplp2*, *Peg10*, *Acyl*, *Cidec*, *Nr3c1*, *Rarres2*, *Car3*, *Selenbp1*, *Nr1d2*, *Ddx17*, *Rybp*, *Adiponectin*, and *Adipsin*), insulin sensitivity-related genes (*Glut4*), and insulin resistance-related genes (*Tnf*, *Ccl2*) in human adipose tissue is analyzed (GSE55200). Log fold changes (log_2_FC) of unhealthy obese individuals (*n* = 8) versus lean healthy individuals (*n* = 7) are calculated. * *p* < 0.05; ** *p* < 0.01; *** *p* < 0.001; **** *p* < 0.0001.
